# Addressing bedaquiline treatment interruptions in the treatment of drug-resistant TB

**DOI:** 10.5588/ijtld.21.0678

**Published:** 2022-07-01

**Authors:** C. Kambili, S. Rossenu, R. M. W. Hoetelmans, E. Birmingham, N. Bakare

**Affiliations:** 1 Johnson & Johnson Global Public Health, New Brunswick, NJ, USA; 2 Janssen Pharmaceutica, Beerse, Belgium; 3 Janssen Research & Development, Titusville, NJ, USA; 4 Johnson & Johnson Global Public Health, Janssen Research & Development, Titusville, NJ, USA

**Keywords:** MDR-TB treatment, BDQ, pharmacokinetics, modelling, dosing

## Abstract

**SETTING:**

The recommended dosing regimen for bedaquiline (BDQ), consisting of a 2-week loading phase (400 mg/day), followed by a maintenance phase (200 mg three times/week), might pose challenges when treatment is interrupted and needs to be reinitiated. Guidance on BDQ treatment re-initiation is, therefore, needed.

**OBJECTIVE:**

This pharmacokinetic-based simulation study aimed to provide recommendations for re-initiating BDQ following treatment interruptions.

**DESIGN:**

Simulations of treatment interruptions, defined as any time a patient misses ≥2 consecutive BDQ doses for up to 56 consecutive days (2 months), were assessed using the BDQ population-pharmacokinetic model.

**RESULTS:**

Any treatment interruption lasting ≤28 days prior to completing the 14-day loading phase can be managed by completing the remaining loading doses. Scenarios when it is sufficient to simply restart maintenance dosing are discussed. In some scenarios, treatment interruptions require reloading for 1 week prior to restarting maintenance dosing.

**CONCLUSIONS:**

This simulation study provided recommendations for managing BDQ treatment interruptions and underscores the importance of having a robust population-pharmacokinetic model for TB drugs to inform clinical guidance. Such recommendations are valuable to help ensure optimal treatment with BDQ for treating multidrug-resistant TB.

## Abstract (fr)

**CONTEXTE:**

Le schéma posologique recommandé pour la bédaquiline, consistant en une phase de charge de 2 semaines (400 mg/jour) suivie d’une phase d’entretien (200 mg trois fois par semaine), peut être problématique lorsque le traitement est interrompu mais qu’il doit ensuite être repris. Des recommandations concernant la reprise du traitement par bédaquiline sont donc nécessaires.

**OBJECTIF:**

Cette étude de simulation pharmacocinétique avait pour objectif de fournir des recommandations pour la reprise de la bédaquiline après interruption du traitement.

**MÉTHODE:**

Les simulations d’interruptions du traitement, définies comme toute période pendant laquelle un patient manque ≥2 doses consécutives de bédaquiline pendant un maximum de 56 jours consécutifs (2 mois), ont été analysées en utilisant un modèle pharmacocinétique de population de la bédaquiline.

**RÉSULTATS:**

Toute interruption de traitement d’une durée de ≤28 jours avant achèvement de la phase de charge de 14 jours peut être compensée en prenant les doses de charge restantes. Des scénarios où il serait juste nécessaire de redémarrer à la posologie du traitement d’entretien sont évoqués. Dans certains scénarios, les interruptions du traitement nécessitent une nouvelle phase de charge d’une semaine avant de recommencer la phase d’entretien.

**CONCLUSIONS:**

Cette étude de simulation fournit des recommandations pour la gestion des interruptions de traitement par bédaquiline et souligne l’importance d’un modèle pharmacocinétique de population robuste pour les antituberculeux afin d’orienter la pratique clinique. De telles recommandations sont précieuses afin de garantir le traitement optimal par bédaquiline de la TB multirésistante.

Lengthy drug-resistant TB (DR-TB) treatment and associated drug toxicities pose significant challenges in ensuring that patients consistently take their medication and complete their treatment as prescribed.^1^,^2^ Patients may not feel the need to continue taking medication as their disease symptoms abate due to effective treatment or due to persistent adverse drug reactions that affect their quality of life. Other reasons for treatment interruptions include a lack of treatment literacy education, lack of resources to procure medicines and ensure a continuous supply, school or work commitments, pill burden, incarceration, and other social factors such as alcohol and drug misuse.^1^

Patients who have interrupted or discontinued treatment will often return for further medical care when TB symptoms recur, work or social situations have stabilised, or adverse events have resolved. As dosing of many TB drugs is generally straightforward, restoring treatment may only require patient education and restarting these drugs. However, certain TB drugs require more complex dosing regimens if treatment is discontinued. Evidence is needed to ensure recommendations on treatment interruptions are included in global and national TB guidelines.

Bedaquiline (BDQ), the first of a new class of antimycobacterial agents that inhibit mycobacterial adenosine triphosphate synthase,^3^ was first approved BDQ as part of a combination therapy for multidrug-resistant TB (MDR-TB) treatment by the US Food and Drug Administration.^4^–^6^ With repeated dosing, BDQ accumulates in tissues and when dosing stops, tissue-bound BDQ is slowly released, accounting for the long terminal elimination half-life of 164 days.^7^ BDQ therefore has a unique dosing schedule of a 2-week loading phase at 400 mg once daily (QD), followed by intermittent dosing at 200 mg three times a week (TIW) for 22 weeks (maintenance phase) in adults and paediatric patients weighing ≥30 kg.^4^ This regimen prevents long-term BDQ accumulation in tissues and maintains the target plasma concentration of ≥600 ng/mL, as selected based on preclinical data.^7^ Previous clinical data showed that most patients achieved average steady-state plasma BDQ concentrations above this target throughout the dosing period.^5^ Discontinuation results in an initial rapid decline in BDQ plasma concentrations.^7^

As BDQ has a unique pharmacokinetic (PK) profile and dosing schedule, re-initiating treatment after an interruption or discontinuation is not straightforward,^4^,^7^ necessitating specific guidance on how to deal with BDQ treatment interruptions that may occur in routine clinical care. A treatment interruption is defined as any time that a patient misses ≥2 consecutive BDQ doses for up to 56 consecutive days (2 months).

The current simulation study aimed to provide a basis for clear recommendations for re-initiating BDQ therapy following treatment interruptions. The objectives were to determine 1) the number of days that a patient may stop taking BDQ without plasma concentrations decreasing below the target concentration of at least 600 ng/mL; and 2) the number of days that a patient may stop taking BDQ but be able to restart the drug by i) completing the remaining loading doses if the break in therapy occurred prior to the end of the loading phase, ii) restarting the 200 mg TIW maintenance dosing scheme when the 14-day loading dose period has been previously completed, or iii) completing an additional 1 week of loading doses.

## METHODS

A population PK model of BDQ developed using PK data from 480 healthy volunteers and patients with DR-TB from nine studies^8^ was used for the current analyses. A series of PK simulations were performed taking into consideration: 1) covariate effects in the population PK model, i.e., race on apparent clearance (CL/F) and sex on apparent volume of distribution for the central compartment (V_c_/F); 2) the uncertainty in parameter estimates of the population PK model; 3) the PK sampling strategy; 4) the exposure to BDQ prior to cessation/interruption of therapy; and 5) the time required for a participant to reach the target of 600 ng/mL after restarting BDQ. Additional information can be found in the Supplementary Data.

As the study was based on an existing population PK model, ethics committee review was not required.

### Simulation scenarios for exposure prior to cessation of therapy

**Figure 1:**
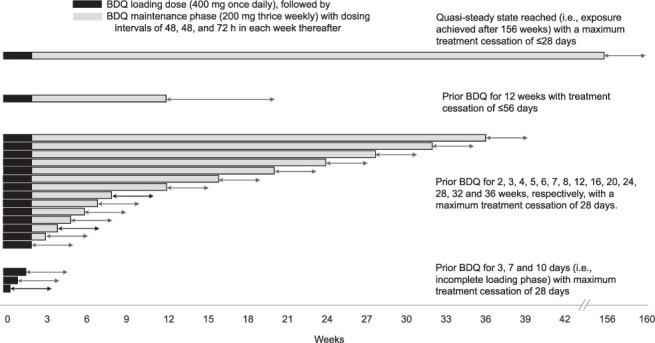
Nineteen scenarios of BDQ exposure periods considered for the simulations. BDQ = bedaquiline.

Nineteen scenarios of BDQ exposure periods were considered for the simulations (Figure 1). Three scenarios were for participants who received prior BDQ for 3, 7 and 10 days (i.e., incomplete loading phase), respectively, with a maximum treatment cessation of 28 days. Fourteen scenarios were for participants who had received prior BDQ for 2, 3, 4, 5, 6, 7, 8, 12, 16, 20, 24, 28, 32 and 36 weeks, respectively, with a maximum treatment cessation of 28 days. One scenario assumed that a quasi-steady state had been reached during the prior exposure period (i.e., the exposure achieved after 156 weeks of treatment according to the therapeutic dosing regimen), with a maximum treatment cessation of ≤28 days. In another scenario, participants received prior BDQ for 12 weeks with treatment cessation of ≤56 days. In each scenario, dosing was assumed to follow the therapeutic dosing regimen of a 2-week loading phase (400 mg QD), followed by a maintenance phase of 200 mg TIW, with dosing intervals of 48, 48 and 72 h within each week thereafter, until the end of each prior exposure period.

### Objective 1: Simulation of time needed to drop below the target BDQ plasma concentration

The median and 95% confidence intervals (CI) of individual simulated plasma concentrations at each PK sampling time point following cessation of BDQ dosing were determined. The time point at which median prediction was <600 ng/mL determined the period a subject may stop taking BDQ without plasma concentrations crossing the concentration exposure cut-point, while the time points at which the 2.5^th^ and 97.5^th^ percentiles crossed 600 ng/mL determined the 95% CI around the cut-off point.

### Objective 2: Simulation of re-initiating treatment with BDQ

Information gained from Objective 1 informed the initial starting points for Objective 2 described below.

#### Treatment restart by completing loading doses

The first scenario assessed the period a subject can stop taking BDQ during the loading phase and be able to restart treatment and complete the remaining loading doses before starting on a maintenance dose of 200 mg TIW. The remaining loading doses followed by two maintenance doses were re-initiated in the dataset assuming a 24-h dosing interval at loading and a 48-h dosing interval at maintenance phase. Simulated concentrations were obtained during the re-initiation period according to a predefined PK sampling strategy (daily sampling times of 0, 1, 3, 5, 6, 8, 12 and 24 h). The median, and 2.5^th^ and 97.5^th^ percentiles for the corresponding average concentration over 48 h (C_ave,48h_) for the two maintenance dosing intervals following treatment restart were computed.

Re-initiation of remaining loading doses started 28 days after cessation of the initial treatment. If median C_ave,48h_ over the second maintenance dosing interval upon re-initiation dropped below 600 ng/mL, the remaining loading doses were re-initiated on Day 27, 26, 25, etc., until the target median (C_ave,48h_) for the second maintenance dosing interval was achieved.

#### Treatment restart on maintenance dosing

The second scenario assessed the period a subject can stop taking BDQ and be able to restart maintenance treatment of 200 mg TIW when the loading phase was previously completed. Two maintenance doses were re-initiated in the dataset assuming a 48-h dosing interval, started on the first day the median predicted BDQ plasma concentration was <600 ng/mL in Objective 1, and at daily time points thereafter for ≤28 days cessation of the initial treatment. Simulated concentrations were obtained during the re-initiation period according to the predefined PK sampling strategy above. The median, and 2.5^th^ and 97.5^th^ percentiles for the corresponding C_ave,48h_ over each of the two dosing intervals following treatment restart were computed.

#### Treatment restart with reloading

The third scenario assessed restarting treatment with the need for a new loading phase. Extending the simulation described above, if the median of the predicted C_ave,48h_ of the second maintenance dosing interval (48–96 h post re-initiation) was below target, i.e., recovery was not achieved within the first two dosing intervals on maintenance dosing for the subject, reloading doses of 400 mg QD for 1 week, followed by two maintenance doses thereafter were included in the dataset and analysed as described above. Simulated concentrations were obtained for the first two maintenance doses following the reloading period according to the predefined PK sampling strategy above. The median, and 2.5^th^ and 97.5^th^ percentiles for the corresponding C_ave,48h_ over each of the two maintenance dosing intervals were computed.

However, if the median of the predicted C_ave,48h_ of the second maintenance dosing interval was below target, reloading for 1 week was considered insufficient and reloading doses of 400 mg QD for 2 weeks would be required.

## RESULTS

### Objective 1: Simulation of time to drop below the target BDQ plasma concentration

For a subject stopping BDQ intake, the shorter the prior exposure period was in each phase (i.e., loading and maintenance), the more rapid the drop in plasma concentrations below the target concentration of 600 ng/mL. During the loading phase, for prior BDQ exposure periods of 3–7 days or 10 days–2 weeks, plasma concentrations dropped below target levels since treatment cessation in <1 or 1 day, respectively (Table 1). During the maintenance phase, for prior BDQ exposure periods of 3–16 weeks or 20–36 weeks, plasma concentrations dropped below target levels in <1 day or 1–13 days, respectively (Table 1).

**Table 1:** Time to drop below target concentration since treatment cessation (first missed dose) in a subject

Prior exposure	Time for BDQ plasma concentration to fall to <600 ng/mL (days) Median (95% CI)
3 days	0 (0–0)
7 days	0 (0–0)
10 days	1 (0–1)
2 weeks	1 (1–3)
3 weeks	0 (0–0)
4 weeks	0 (0–0)
5 weeks	0 (0–0)
6 weeks	0 (0–0)
7 weeks	0 (0–0)
8 weeks	0 (0–1)
12 weeks	0 (0–4)
16 weeks	0 (0–11)
20 weeks	1 (0–19)
24 weeks	3 (0–>28)
28 weeks	6 (0–>28)
32 weeks	10 (0–>28)
36 weeks	13 (0–>28)
QSS^†^	41 (0–>57)

* 2.5th percentile–97.5th percentile.

† QSS: 156 weeks of prior exposure.

BDQ = bedaquiline; CI = confidence interval; QSS = quasi-steady-state.

### Objective 2: Simulation of re-initiating treatment with BDQ

#### Treatment restart by completing loading doses

**Figure 2:**
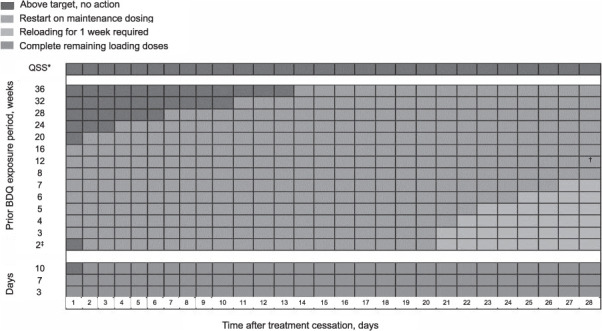
A nomogram summarising the recommended approach for re-initiation of BDQ therapy following treatment interruptions of ≤28 days. *QSS: 156 weeks of prior exposure. ^†^Assessed out to 56 days: days 1–≤39 = restart on maintenance dosing, days ≥40–≤56 =reloading for 1 week required. ^‡^Two-week loading phase of BDQ at 400 mg once daily. QSS =quasi-steady-state; BDQ = bedaquiline.

For a subject, target plasma concentrations (≥600 mg/ml) were achieved for all simulation scenarios assuming prior exposure periods of 3, 7 and 10 days during the BDQ loading phase when loading doses were re-initiated for ≤28 days post cessation prior to completing the 14-day loading phase (Table 2A and Figure 2).

**Table 2:** Plasma concentrations during the second maintenance dosing interval following 1) completion of loading dose and maintenance treatment initiation; 2) maintenance treatment restart; and 3) 1 week of dose reloading

Prior exposure	Restart of dosing (day)	BDQ plasma concentration (ng/mL) Median (95% CI)*
Completing loading doses, days^†^		
3	28	777.5 (659.9–883.2)^§^
7	28	725.8 (611.9–827.0)^§^
10	28	679.4 (571.1–778.1)^§^
Restart of maintenance dosing, weeks^‡^		
2	20	601.8 (502.7–692.3)^§^
2	21	595.9 (497.9–685.4)^¶^
3	20	602.6 (500.3–695.7)^§^
3	21	597.4 (495.7–689.9)^¶^
4	21	601.8 (492.7–699.2)^§^
4	22	595.9 (487.8–692.3)^¶^
5	22	602.7 (493.0–703.3)^§^
5	23	598.2 (489.0–698.3)^§^
6	24	601.7 (488.4–704.9)^§^
6	25	597.4 (484.6–700.3)^¶^
7	26	600.9 (485.4–708.0)^§^
7	27	596.9 (481.9–703.9)^¶^
8	28	601.8 (483.0–713.4)^§^
12	28	632.7 (502.7–765.1)^§^
16	28	671.8 (523.2–812.4)^§^
20	28	706.3 (544.6–854.1)^§^
24	28	735.1 (561.2–897.8)
28	28	765.1 (572.5–934.5)^§^
32	28	788.4 (584.1–962.9)^§^
36	28	804.3 (595.9–992.3)^§^
12	39	601.2 (473.8–723.4)^§^
12	40	598.4 (471.4–720.1)^¶^
Restart with 1 week of reloading dosing, weeks^#^		
2	28	854.1 (713.4–982.4)
3	28	862.6 (713.4–992.3)
4	28	871.3 (720.5–1002.2)
5	28	871.3 (720.5–1012.3)
6	28	880.1 (720.5–1022.5)
7	28	888.9 (727.8–1032.8)
12	56	871.3 (692.3–1022.5)

* 2.5th percentile–97.5th percentile.

† Represents the day of loading dose treatment re-initiation post cessation of dosing.

‡ Represents the day that maintenance treatment was restarted post cessation of dosing:

§ indicate that the target was reached;

¶ indicate that the target was not reached.

# Represents the day that treatment was re-initiated with once-daily loading doses for 1 week post cessation of dosing; maintenance dosing commences 7 days after the initiation of reloading: values indicate that the target was reached for maintenance dosing following 1 week of reloading. BDQ = bedaquiline; CI = confidence interval.

#### Treatment restart on maintenance dosing

For a subject stopping BDQ and re-initiating maintenance treatment of 200 mg TIW when the loading dose was previously completed, target plasma concentrations (≥600 mg/ml) were achieved when restarting maintenance dosing 20, 20, 21, 22, 24 or 26 days post cessation after prior exposure periods of 2, 3, 4, 5, 6 and 7 weeks, respectively. Target plasma concentrations were also achieved assuming prior exposure periods ≥8 weeks when maintenance treatment was re-initiated for ≤28 days post cessation and for 12 weeks’ prior exposure when maintenance treatment was re-initiated for ≤39 days post cessation (Table 2B and Figure 2).

#### Treatment restart with reloading

Target plasma concentrations (≥600 mg/ml) were not achieved following maintenance treatment re-initiation on 21, 21, 22, 23, 25, 27 and 40 days post cessation for prior BDQ exposure periods of 2, 3, 4, 5, 6, 7 and 12 weeks, respectively.

Further simulations showed 1 week of reloading with 400 mg BDQ QD initiated on Day 28 post-treatment cessation for prior exposure periods of 2, 3, 4, 5, 6 and 7 weeks and on Day 56 post-treatment cessation for a prior exposure period of 12 weeks, respectively, achieved target plasma concentrations of ≥600 ng/mL, at the maintenance treatment, following the reloading period (Figure 2 and Table 2C).

## DISCUSSION

It is well recognised that completion of therapy, as recommended in guidelines, has been challenging for TB, as treatment is long.^9^–^11^ A broad range of approaches to achieve treatment adherence and completion should be integral to the treatment plan for all forms of TB.^12^ The need to complete therapy is even greater for DR-TB treatment.^9^–^12^ Treatment adherence with anti-TB drugs is critical to achieving good outcomes, including cure of individual patients, controlling disease spread and preventing drug resistance development.^12^ The WHO and other agencies now recommend ‘patient-centred approaches’ to support patients with TB in achieving favourable outcomes.^10^,^13^ Restoring interrupted or discontinued treatment is a key factor in the fight against TB, and restarting treatment will depend upon the particular drug and its dosing regimen.

Guidance on managing individual missed doses is provided in current BDQ labelling,^4^ which states that if a BDQ dose is missed during the loading phase, patients should not make up the missed dose but continue the usual dosing schedule. If a dose is missed during the maintenance phase, patients should take the missed dose and adjust the dosing schedule to ensure the total dose during the 7-day period does not exceed 600 mg (taken as three intakes of 200 mg per day, ≥24 h apart).

Our findings support label recommendations for interruptions at loading phase and provide more granular recommendations beyond the scenario of individual missed doses for re-initiating BDQ therapy following maintenance phase interruptions. For treatment interruptions ≤28 days prior to loading phase completion, the patient should simply resume intake of any remaining loading doses to complete 14 days of loading, prior to starting on a maintenance dose of 200 mg TIW. For treatment interruptions ≤20 days during the maintenance phase with 2–7 weeks of prior BDQ exposure, treatment interruptions ≥21–≤28 days (≥8 weeks of prior BDQ exposure) and ≤39 days (12 weeks of prior BDQ exposure), the patient can simply resume maintenance dosing. Treatment interruptions of ≥21–≤28 days and ≥40–≤56 days during the maintenance phase after 2–7 or 12 weeks of prior BDQ exposure, respectively, require reloading for 1 week at 400 mg daily prior to resuming maintenance dosing at 200 mg TIW. These evidence-based recommendations will help physicians and their patients to ensure, following interruption or discontinuation, BDQ treatment can be restarted appropriately.

Although two further simulation studies have been performed based on only BDQ and M2 data from patients with MDR-TB in the Phase 2 BDQ studies,^14^–^16^ we consider our simulation study to be more robust. Our findings are based on the approved dosing regimen for BDQ, for the approved treatment duration of ≤24 weeks (6 months), and on the population PK model for BDQ in a larger cohort of subjects with rich and sparse PK sampling (5,222 PK observations from 480 participants in nine studies, including both healthy volunteers and TB-infected patients, compared with 2,843 PK observations from 335 participants in two studies).^8^,^14^ Nevertheless, all three studies support each other to some degree. We observed consistency in that interruptions of ≤4 weeks after week 1 of BDQ treatment require completion of a 1-week BDQ 400 mg QD loading dose, and that interruptions <2 weeks following 4–16 weeks BDQ exposure only require restarting with maintenance phase dosing.^15^,^16^ For treatment interruptions ≤2 months following 2–36 weeks of prior BDQ exposure during the maintenance phase, our analysis provides more detailed recommendations by simulating scenarios for re-initiating with BDQ maintenance dosing (in some cases necessitating reloading for 1 week), rather than just evaluating re-starting with a 400 mg QD reloading dose in all scenarios.

As our data and subsequent recommendations are based on simulation, limitations should be considered. We have only provided information for BDQ, while patients with DR-TB are treated with combination of drugs, although our population PK model did include data from patients receiving multiple drug combinations in BDQ clinical trials.^8^ Information on the PK or pharmacodynamic characteristics of many of these other drugs is sparse, as they have not undergone rigorous testing in a controlled setting. The most appropriate use of BDQ, as guided herein, may therefore not be enough to counteract the multiplicity of pharmacological factors that can impact DR-TB treatment outcomes. Nonetheless, we believe that our current observations can be applied to most patients with DR-TB undergoing BDQ treatment, as it would not be feasible to ‘individualise’ our recommendations. It should be noted that this model only looks at the currently approved BDQ dosing and does not evaluate any other dosing paradigms, including daily dosing regimens in ongoing trials and the reduced dose recommended for children by the WHO.^11^ As with any simulation study, real-world examination of these recommendations would be beneficial.

We acknowledge M2 exposures were not simulated in our analysis, and since high M2 exposure may increase risk of QTc-interval prolongation, this is a possible limitation. However, although M2 plasma concentrations have been implicated with QT prolongation, no clear relationship between BDQ or M2 plasma level and corresponding absolute QTcF values or changes in the QTcF interval have been clinically demonstrated.^6^ While a recent exposure-safety model of data from two Phase IIb studies found increased M2 concentration may cause treatment-associated QTc-interval prolongation, this has not been validated clinically. This model also predicted doses of BDQ above the approved dose will not lead to critical QTcF interval increases.^17^ We consider our simulations for BDQ (parent compound) exposure appropriate for efficacy and safety.

## CONCLUSIONS

TB treatment interruption can result in unfavourable outcomes and emergence of drug resistance. Based on simulations, recommendations on managing BDQ treatment interruptions can be made. Our study underscores the importance of a robust PK dataset and associated PK model for TB drugs on which clinical guidance can be based. For BDQ, with its unique dosing scheme (consisting of a 2-week loading phase, followed by a maintenance phase), the current simulation analyses support guidance to ensure optimal treatment with BDQ in response to treatment interruptions.
